# Neuroprotective effects of luteolin against aluminum-induced brain injury via chelation and antioxidant pathways

**DOI:** 10.1007/s00210-026-05459-7

**Published:** 2026-05-27

**Authors:** Şeyma Aydın, Elif Dalkılınç, Selçuk Özdemir, Sefa Küçükler, Selim Çomaklı, Ahmet Adıgüzel

**Affiliations:** 1 Department of Molecular Biology and Genetics, Faculty of Science, Atatürk University, Erzurum, Türkiye; 2 Department of Biochemistry, Faculty of Veterinary Medicine, Atatürk University, Erzurum, Türkiye; 3 Department of Genetics, Faculty of Veterinary Medicine, Atatürk University, Erzurum, Türkiye; 4 Department of Pathology, Faculty of Veterinary Medicine, Atatürk University, Erzurum, Türkiye

**Keywords:** Aluminum, Luteolin, Neurodegeneration, Oxidative stress, Apoptosis, Inflammation

## Abstract

Aluminum (Al) is a widespread environmental neurotoxin linked to several neurodegenerative conditions through oxidative stress, inflammation, and apoptosis. Because it is present in food, water, pharmaceuticals, and consumer products, chronic exposure is nearly unavoidable and poses a public health concern. Luteolin (LUT), a natural flavone with antioxidant, anti-inflammatory, and metal-chelating properties, has emerged as a potential neuroprotective agent. This study investigated the protective effects of LUT against aluminum chloride (AlCl_3_)-induced neurotoxicity in rats. Sprague–Dawley rats received AlCl_3_ (4.2 mg/kg, i.p.) alone or together with LUT (25 or 50 mg/kg, p.o.) for 30 days. Behavioral performance was evaluated using novel object recognition and open-field tests. ICP-MS was employed to quantify brain Al levels, and complementary analyses were conducted to assess oxidative stress, neuroinflammation, apoptosis-related gene expression, neuroplasticity markers (BDNF, c-Fos), and cholinergic function (AChE). AlCl_3_ exposure significantly increased brain Al accumulation and induced oxidative stress, inflammation, apoptosis, and behavioral deficits. Although LUT co-treatment led to an apparent increase in total brain Al, this rise is likely attributable to the formation of Al–LUT complexes. This interpretation is supported by the marked reduction in Al-induced oxidative stress, apoptosis, and inflammation, accompanied by improved behavioral outcomes. This evaluation reveals that LUT provides protection in an environmental Al neurotoxicity model, offering insights beyond classical Alzheimer’s disease contexts. Although its antioxidant and anti-inflammatory effects are well documented, evidence for its efficacy against environmentally induced Al neurotoxicity is still lacking.

**Graphical Abstract:**

**Figure:**
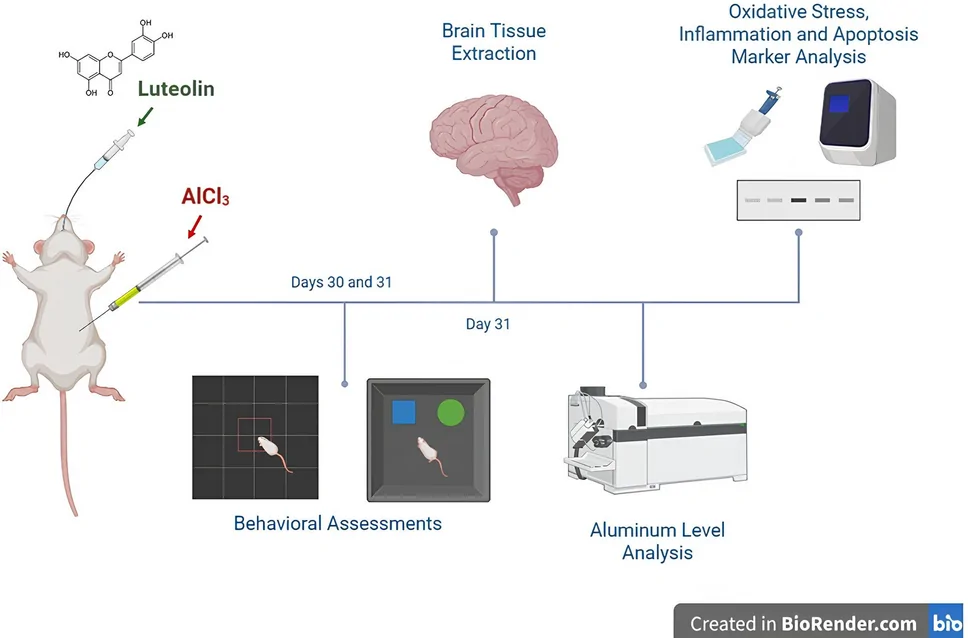

**Supplementary Information:**

The online version contains supplementary material available at 10.1007/s00210-026-05459-7.

##  Introduction

Aluminum (Al) is a naturally occurring metal found in soil, water, and air; however, its environmental concentrations have significantly increased in recent decades due to human activities such as improper waste disposal, industrial manufacturing, and mining (Fuge 2013; Skibniewska and Skibniewski 2019). Humans are therefore continuously exposed to Al through a variety of sources, including contaminated drinking water, foods, food additives, pharmaceutical products, cosmetic items, and Al-containing cookware (Stahl et al. 2018).

The primary route of Al entry into the human body is oral ingestion, with the average daily dietary intake typically ranging from 4 to 9 mg (Yokel et al. 2008). In some developing countries, Al leaching from cookware can reach alarmingly high levels of up to 125 mg per serving, exceeding the World Health Organization’s recommended daily limit of 20 mg per person (Weidenhamer et al. 2017). Furthermore, the use of non-prescription antacids and anti-ulcer medications containing aluminum salts, such as aluminum hydroxide, has been implicated as a significant source of Al exposure in humans, especially when taken at high doses or over prolonged periods (Igbokwe et al. 2019). Excessive Al exposure poses significant health risks, as aluminum ions have no known physiological function in the human body and once absorbed, can accumulate and induce damage in various tissues, including the liver, kidneys, and brain (Niu 2023). However, among the tissues mentioned, the brain is the most sensitive, as neurons are highly vulnerable and possess only a limited capacity for regeneration (Cirovic et al. 2023).

Al can exert toxic effects in the brain by crossing the blood–brain barrier (BBB) through diffusion, carrier-mediated, and receptor-mediated pathways, with receptor-mediated transport, particularly via the transferrin receptor 1 (TfR1)-Al complex, being the predominant route (Wang 2023). This allows Al to bypass protective mechanisms and accumulate throughout the brain (Choudhury et al. 2023). Over time, its buildup may trigger chronic neuroinflammation, alter neuronal gene expression, cause irreversible neuronal damage, and lead to motor, behavioral, and cognitive impairments (Inan-Eroglu and Ayaz 2018).

The neurodegenerative effects of Al are thought to result from mechanisms such as excessive microglial activation, disruption of neurotransmitter balance, abnormal cytokine production, and increased oxidative stress (Pankaj Bhargava et al. 2021; Makhdoomi et al. 2023). Furthermore, chronic Al exposure has been suggested to disrupt metal homeostasis and promote the formation of insoluble Al complexes that damage vital biomolecules such as proteins, lipids, and DNA (Saeed et al. 2021; Zhao et al. 2022b). Specifically, Al’s affinity for DNA phosphate groups may induce structural changes that contribute to genomic instability (Dey and Singh 2022a).

Several studies have examined the potential relationship between Al and neurodegenerative disorders. Exley and Clarkson (2020), through post-mortem examinations, reported elevated brain Al levels in patients with Alzheimer’s disease (AD), multiple sclerosis (MS), and autism compared to healthy individuals. A similar study analyzing 511 human brain samples from 18 neurological and neurodegenerative disorders, including age-matched controls, reported significantly higher Al concentrations in AD, Down syndrome, and dialysis dementia syndrome, whereas no clear association was observed in other CNS conditions (McLachlan et al. 2018). In another study, Linhart et al. measured brain Al levels in donors without MS and compared them with MS cases. Although Al was present in all donors and increased with age, significantly higher levels were observed in MS brains, supporting its potential contribution to MS etiology (Linhart et al. 2020). Overall, these observations point to a potential but inconsistent association between Al and neurodegeneration, highlighting the importance of further research to elucidate underlying mechanisms and to develop effective therapeutic strategies targeting this process.

Diets rich in polyphenols and flavonoids have been associated with reduced risk of neuroinflammatory and neurodegenerative conditions (Calis et al. 2020). Luteolin (LUT), a naturally occurring flavone, exhibits potent antioxidant and anti-inflammatory properties and is capable of crossing the BBB, thereby exerting direct neuroprotective effects (Huang et al. 2023; Pradhan and Kulkarni 2025). Experimental evidence indicates that LUT suppresses glial activation, attenuates reactive oxygen species (ROS) generation, and modulates pro-inflammatory signaling pathways in the central nervous system (Kempuraj et al. 2021).

However, most studies have evaluated LUT within classical neurodegenerative or injury models. In contrast, Al-induced neurotoxicity represents a distinct condition characterized by progressive metal accumulation and metal-dependent molecular dysregulation. Whether LUT can modulate Al complexation and metal-triggered signaling cascades remains insufficiently understood. Therefore, the present study employed a chronic AlCl_3_ exposure model to investigate the mechanistic basis of LUT-mediated neuroprotection through integrated behavioral, biochemical, and molecular analyses.

In this context, AlCl_3_ was employed to mimic chronic exposure to an environmental neurotoxicant, allowing the investigation of LUT’s potential protective role against Al-induced brain injury. A comprehensive experimental approach was employed, including behavioral assessments, biochemical analyses of oxidative stress and inflammatory markers, quantification of metal accumulation, and investigation of molecular pathways implicated in neurodegeneration, to clarify LUT’s therapeutic potential and contribute to the development of effective interventions.

## Materials and methods

### Chemicals

Luteolin (LUT) was obtained from BLD Pharm (Cat. No. BD6331, Purity 98%, China), and AlCl_3_ anhydrous was purchased from Carlo ERBA (Cat. No. 416996, Purity: 99%, Germany).

### Animals

Thirty-five adult male Sprague–Dawley rats, weighing 250–300 g at 10 weeks of age, were purchased from Ataturk University’s Experimental Research and Application Center (ATADEM). The animals were acclimated for a week in controlled conditions, which included a 12-h light/dark cycle, a temperature of 24 ± 1 °C, and a relative humidity of 45 ± 5%. The rats received full access to tap water and standard laboratory chow during the acclimation and experimental phases. The Ataturk University Local Ethics Committee for Animal Experiments gave its approval for all procedures, which were carried out in compliance with ethical guidelines (approval no. 2024–02/30).

### Groups and design of the study

Experimental animals were randomly divided into five groups (*n* = 7 per group): control, LUT, AlCl_3_, AlCl_3_ + LUT25, and AlCl_3_ + LUT50. The control group received sterile physiological saline intraperitoneally (i.p.) once daily for 30 days. The LUT group received 50 mg/kg body weight of LUT by oral gavage once daily for 30 days, with the dose adapted from a previous study (Jiang et al. 2022). The AlCl_3_ group received 4.2 mg/kg/bw AlCl_3_ i.p. once daily for 30 days, according to a previous report that demonstrated neurotoxicity at this dose (Turkez et al. 2022). The AlCl_3_ + LUT25 group received AlCl_3_ (4.2 mg/kg/bw, i.p.) together with LUT (25 mg/kg/bw, orally) once daily for 30 days, while the AlCl_3_ + LUT50 group received the same dose of AlCl_3_ in combination with LUT (50 mg/kg/bw, orally) for the same period.

### Behavioral evaluation

Open field test (OFT) and novel object recognition (NOR) test were performed on days 30 and 31 to evaluate locomotor activity, anxiety-/depression-like behaviors, and memory impairment. All tests were recorded using a specialized software program (ANY-maze, Ireland) that tracked the animals’ movements.

#### OFT for evaluation of locomotor behavior and anxiety-like behavior

OFT was performed using a modified method based on Abbas et al. (2022). A wooden arena measuring 100 × 100 cm with 40-cm-high walls and a dark-colored floor was used. Each rat was individually placed in the center of the arena, and its behavior was video recorded for 5 min under standard lighting conditions. ANY-maze software automatically quantified the total distance travelled and the time spent in the central nine squares versus the peripheral 16 squares. To avoid olfactory bias, the arena was thoroughly cleaned with 70% ethanol after each session.

Each experimental group initially consisted of seven animals. However, during the OFT, some rats exhibited no exploratory behavior (i.e., complete immobility), which rendered their data unreliable. Therefore, data analysis was performed on five animals per group.

#### Evaluation of short and long-term memory using the NOR test

Recognition memory was assessed using the NOR test, adapted from the methodology of Salimzade et al. (2017) with slight modifications. The same open-field apparatus used in the OFT was employed for the NOR procedure. Since all animals were already exposed to the apparatus during the OFT, this session served as the habituation phase; therefore, no additional separate habituation was conducted. The procedure consisted of a training session, in which rats were exposed to two identical objects for 5 min, followed by a test session in which one of the familiar objects was replaced with a novel one. Exploration was defined and recorded automatically by the ANY-maze software, which detected the nose-point of the animal within a ≤ 2 cm zone drawn around each object. Behavioral activity was quantified as the time spent exploring the novel versus the familiar object, and recognition memory performance was evaluated based on these measures. From this data, two key measures were derived: the recognition index, indicating memory retention of objects, and the discrimination index, reflecting the ability to distinguish between novel and familiar objects.

Recognition index (RI):

$$RI=\frac{Time\;spent\;on\;novel\;object}{Time\;spent\;on\;novel\;object+Time\;spent\;on\;familiar\;object}$$

Discrimination index (DI):

$$DI=\frac{Time\;spent\;on\;novel\;object-Time\;spent\;on\;familiar\;object}{Time\;spent\;on\;novel\;object+Time\;spent\;on\;familiar\;object}$$

The DI varies between − 1 and 1, with − 1 indicating a strong preference for the familiar object, 0 indicating no preference, and 1 representing a strong preference for the new object.

### Preparation of brain specimens

At the end of the experimental period and following behavioral assessments, rats were euthanized under mild sevoflurane anesthesia. The whole brain was carefully excised, cleared of adhering tissues and washed with cold phosphate-buffered saline. Each brain was then divided along the midline into two hemispheres: one hemisphere was fixed in 10% formaldehyde for histopathological evaluation, while the other was flash-frozen and stored at − 80 °C for molecular and biochemical analyses.

### Determination of Al contents in brain tissue by ICP-MS analysis

Brain Al levels were measured using an Agilent 7800 ICP-MS. 300 mg of whole brain tissue was digested with 5 mL of ultrapure nitric acid and 2.5 mL of hydrogen peroxide in a closed-vessel microwave digestion system. After digestion, the solutions were evaporated, diluted to 10 mL with high-purity water, and stored at 4 °C until analysis. Samples were introduced into the ICP-MS with a scandium internal standard (40 µg/L) to correct for matrix effects. Calibration was performed using Al standard solutions containing the internal standard, and results were expressed as µg Al per g wet tissue.

### UV–Vis spectrophotometric evaluation of LUT–Al chelation

The chelation interaction between LUT and AlCl_3_ was evaluated using ultraviolet–visible (UV–Vis) spectrophotometry according to the method described by Sikora and McBride (1989). To create a stock solution with a concentration of 0.01 mg/mL, LUT was dissolved in methanol. Three milliliters of this LUT solution was combined with 0.3 ml of 0.1 mol/L AlCl_3_ solution for the chelation test. The mixture was then treated with 0.15 mL of 12% hydrochloric acid (HCl) to create an acidic environment that would support the formation of complexes. The resulting solutions were immediately subjected to absorbance measurements using a UV–Vis spectrophotometer (Shimadzu, Japan), scanning across the wavelength range of 200 to 500 nm. Changes in the absorbance spectra were analyzed to determine the extent and nature of the complex formed between LUT and AlCl_3_, indicating the chelating ability of LUT under these conditions.

### Biochemical evaluation of oxidative damage and antioxidant defense in brain homogenates

Brain tissue homogenates were prepared and used to evaluate key antioxidant enzyme activities and markers of oxidative damage. Superoxide dismutase (SOD) activity in brain homogenates was measured using a colorimetric assay adapted from Sun et al. (1988). Catalase (CAT) activity was evaluated by applying the spectrophotometric method originally developed by Aebi (1984). Glutathione peroxidase (GPx) activity was determined according to the enzymatic assay protocol described by Matkovics et al. (1988). These activity-based assays were selected to evaluate functional antioxidant capacity. The concentration of reduced glutathione (GSH) was quantified based on the spectrophotometric technique introduced by Sedlak and Lindsay (1968). Lipid peroxidation was assessed by measuring malondialdehyde (MDA) levels following Placer et al. (1966). Although more specific chromatographic methods (e.g., HPLC-based assays) are available for MDA determination, the TBARS spectrophotometric method was selected due to its widespread use in experimental neurotoxicity studies and suitability for standardized comparative group analyses. Total protein content was determined by the Lowry method (Lowry et al. 1951) to normalize enzyme activities and oxidative stress markers.

### Measurement of c-Fos, AChE, and BDNF levels in brain tissue using ELISA

The concentrations of cellular oncogene Fos (c-Fos), acetylcholinesterase (AChE), and brain-derived neurotrophic factor (BDNF) in brain tissue samples were quantified utilizing commercially available ELISA kits specifically designed for rats (Sunred, Shanghai, China). The catalog numbers and detection ranges for the kits were as follows: c-Fos (Cat. No. 201–11–8477; detection range 0.3–30 ng/mL), AChE (Cat. No. 201–11–0725; detection range 0.3–30 ng/mL), and BDNF (Cat. No. 201–11–0477; detection range 0.04–10 ng/mL).

Frozen brain tissues were thawed and homogenized in PBS (pH 7.4) at a 1:20 (w/v) ratio. After homogenization, samples were centrifuged as per the manufacturer’s guidelines to obtain clear supernatants. These supernatants were then used in ELISA assays following the kit protocols, and protein levels of c-Fos, AChE, and BDNF were quantified based on standard curves.

### Expression analysis of Bax, Bcl-2, Nrf2, HO-1, NF-κB, p53, and JNK using real-time PCR

As directed by the manufacturer, total RNA was extracted from 50 mg of brain tissue using QIAzol Lysis Reagent (Qiagen, Cat. No. 79306, Germany). The BioTek Epoch NanoDrop® spectrophotometer was used to measure the concentration and purity of the extracted RNA. Then, following the recommended protocol, complementary DNA (cDNA) was synthesized from the purified RNA samples using the iScript cDNA Synthesis Kit (Bio-Rad, Cat. No. 4368814).

Using the QuantiNova® SYBR® Green PCR Kit (Qiagen, Cat. No. 208056, Germany), quantitative real-time PCR (qRT-PCR) was carried out on a Rotor-Gene Q 5plex HRM Platform (Qiagen, Germany) in order to analyze gene expression. The following primers (primer sequences listed in Table 1) were used in triplicate reactions: Bax (Bcl-2-associated X protein), Bcl-2 (B-cell lymphoma 2), p53 (Tumor Protein 53), NF-κB (Nuclear factor kappa-light-chain-enhancer of activated B cells), JNK (c-Jun N-terminal kinase), Nrf2 (Nuclear Factor Erythroid 2-Related Factor 2), and HO-1 (Heme Oxygenase 1). The 2^−ΔΔCt^ method was used to calculate relative gene expression levels across samples after normalizing expression data to the housekeeping gene β-actin (Livak and Schmittgen 2001).

**Table 1:** Nucleotide sequences of primers employed for quantitative analysis of gene expression via real-time polymerase chain reaction (qRT-PCR)

Name	Accession number	Forward primer (5′−3′)	Reverse primer (5′−3′)
B-actin	NM_031144.3	GGAGATTACTGCCCTGGCTCCTAGC	GGCCGGACTCATCGTACTCCTGCTT
Bax	NM_017059.2	CCAGGACGCATCCACCAAGAAGC	TGCCACACGGAAGAAGACCTCTCG
Bcl-2	NM_016993.2	TATATGGCCCCAGCATGCGA	GGGCAGGTTTGTCGACCTCA
Nrf-2	NM_001427235.2	TCAGCTACTCCCAGGTTGCCCA	GGCAAGCGACTCATGGTCATCTAC
HO-1	NM_012580.2	CACATCCGTGCAGAGAATTCT	TTCGCTCTATCTCCTCTTCCAG
NF-κb	NM_001415012.1	CAGCACTCCTTATCAACCACC	CTCCTGAGCGTTGACTTCTG
p53	NM_001429996.1	GTCGGCTCCGACTATACCACTATC	CTCTCTTTGCACTCCCTGGGGG
JNK	XM_039109986.1	CCAGGACTGCAGGAACGAGT	CCACGTTTTCCTTGTAGCCC

The abbreviations of the genes: *B-actin*, Beta Actin; *Bax*, Bcl-2-associated X protein; *Bcl-2*, B-cell lymphoma gene-2; *Nrf-2*, Nuclear Factor Erythroid 2-Related Factor 2; *HO-1*, Heme oxygenase 1; *NF-κb*, Nuclear factor-κB; *p53*, Tumor Protein 53; *JNK*, c-Jun N-terminal kinase

### Protein isolation and western blot analysis

Fifty milligrams of brain tissue was homogenized in 500 µL of RIPA buffer (Santa Cruz, Cat. No. sc-24948) and centrifuged at 1400 × *g* for 30 min at 4 °C to perform Western blot analysis. Protein concentrations were measured using the Pierce™ BCA Protein Assay Kit (Thermo Fisher, Cat. No. A55864) after supernatants were collected. Using the Trans-Blot® Turbo™ Transfer System (Bio-Rad), equal amounts of protein (50 µg) were loaded onto 12% SDS-PAGE gels and then transferred to PVDF membranes.

After blocking the membranes with 5% skim milk in TBS-T for 1 h at room temperature, the membranes were incubated with primary antibodies against IL-1β (Santa Cruz, Cat. No. sc-52012, RRID: AB_629741) and GAPDH (Santa Cruz, Cat. No. sc-32233, RRID: AB_627679) at 4 °C for 12 h. Following washing steps, membranes were incubated with an HRP-conjugated secondary antibody (Santa Cruz, Cat. No. sc-2005, RRID: AB_631736; 1:5000 dilution) for 1 h at room temperature. Protein bands were detected using Clarity™ Western ECL substrate (Bio-Rad, Cat. No. 1705061), visualized with a ChemiDoc™ MP imaging system (Bio-Rad), and quantified using ImageJ software.

### Histopathological processing

After the study, brain tissues from all animals were washed with cold phosphate-buffered saline and then fixed in 10% formalin. After trimming the tissues and removing formaldehyde, they were processed through graded alcohols (70%, 80%, 96%, 100%) and xylene, and then embedded in paraffin. Paraffin sections with a thickness of 5 µm were taken onto the normal slide. For deparaffinization, the sections were incubated at 57 °C for 1 h and then passed through xylene. For rehydration, sections were passed through a decreasing series of ethanol concentrations (100%, 90%, 70%, and 50%, each for 5 min). Afterwards, the sections were immersed in hematoxylin for 2 min, washed under running tap water, and counterstained with eosin for 1 min. To dehydrate the sections, they were passed through a graded increasing ethanol series (50%, 70%, 90%, and 100%, each for 5 min) and then immersed in xylene before coverslipping. After staining, histopathological changes were examined using a light microscope under × 10 magnification. Each section was scored on a scale of 0 to 3 as follows: no damage (0), mild (1), moderate (2), and severe (3).

The Kruskal–Wallis test, followed by the Mann–Whitney *U* test, was used to evaluate differences in histopathological findings between groups.

### Statistical analysis

Data were analyzed using GraphPad Prism (version 9.5.0; GraphPad Software, San Diego, CA, USA). Normality and homogeneity of variances were confirmed by the Shapiro–Wilk and Brown-Forsythe tests, respectively. Data with normal distribution and equal variances were analyzed by one-way ANOVA followed by Tukey’s post hoc test. Results are presented as mean ± SEM, with significance set at *p* < 0.05. In figures, significance between control and AlCl_3_-treated groups is indicated as ns (*p* > 0.05), *p < 0.05, **p < 0.01, ***p < 0.001, and ****p < 0.0001.

## Results

### Behavioral tests

#### Effects of LUT on AlCl_3_-induced alterations in anxiety-like behavior and locomotor activity

**Fig. 1:**
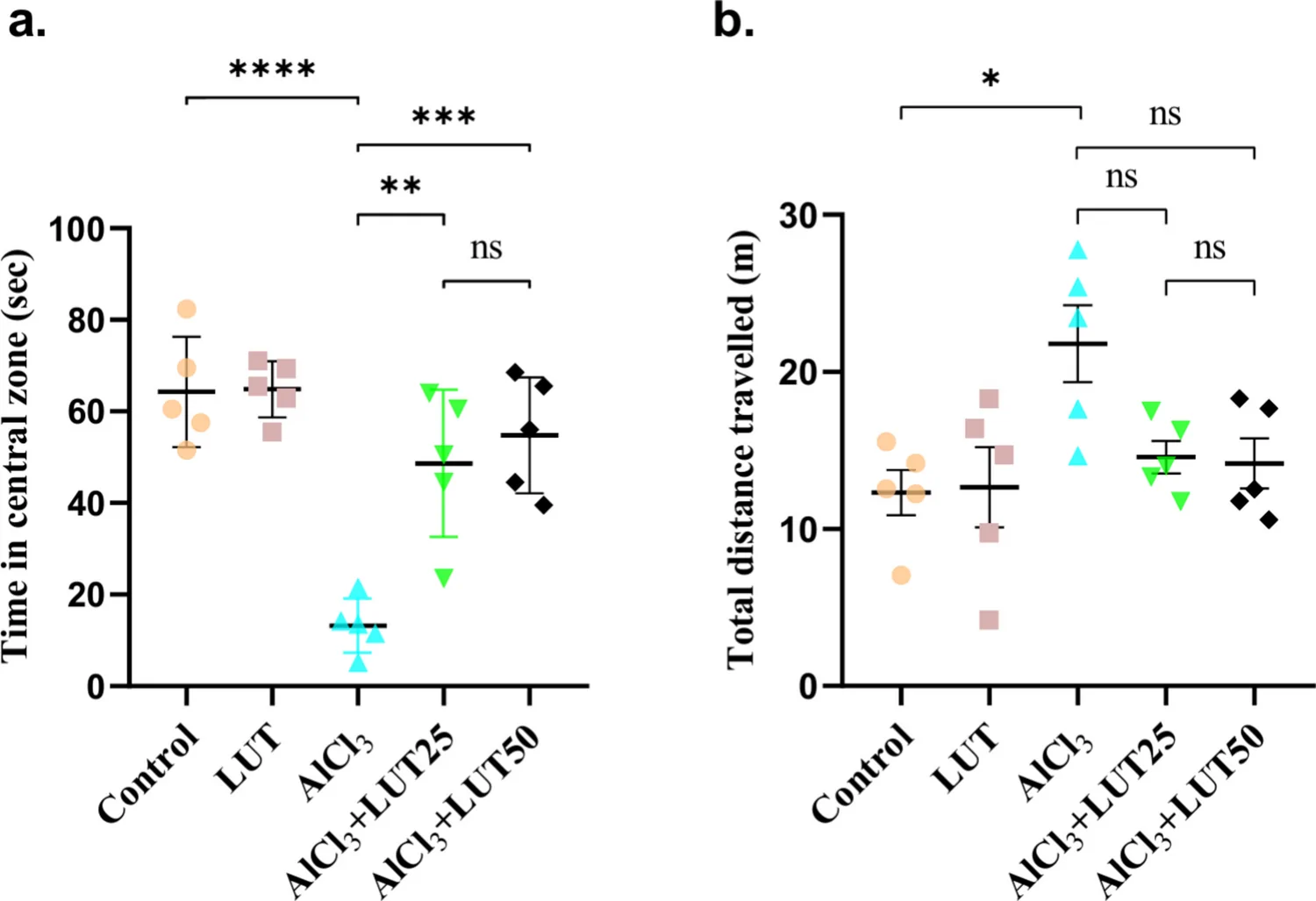
**a** Time spent in the central zone (sec), **b** total distance traveled (m). Data are presented as mean ± SEM (*n* = 5 per group). Statistical significance is indicated as: ns, not significant; *p < 0.05; **p < 0.01; ***p < 0.001; ****p < 0.0001

In the OF arena, rats in the AlCl_3_ group exhibited a marked reduction in central zone exploration during the 5-min observation period, spending significantly less time in the center than the control group (*p* < 0.0001, Fig. 1a). In contrast, rats in the AlCl_3_ + LUT25 and AlCl_3_ + LUT50 groups spent significantly more time in the central area relative to the AlCl_3_ group (*p* < 0.01 and *p* < 0.001, respectively), indicating attenuation of anxiety-like symptoms. However, there was no significant difference between the two LUT-treated groups. These findings indicate that LUT treatment mitigates AlCl_3_-induced anxiety-like behavior by promoting exploration of the central zone.

Regarding locomotor activity (Fig. 1b), AlCl_3_ exposure resulted in higher total distance travelled compared with the control group (*p* < 0.05). No significant differences were observed between the AlCl_3_ and LUT co-treatment groups or between the two LUT doses (*p* > 0.05). To further clarify the behavioral interpretation, the distribution of time and distance between the central and peripheral zones was also examined (see Supplementary File S1). These parameters similarly indicated reduced center exploration in the AlCl_3_ group and partial normalization following LUT treatment.

#### LUT treatment reverses AlCl_3_-induced recognition memory deficits in the NOR test

**Fig. 2:**
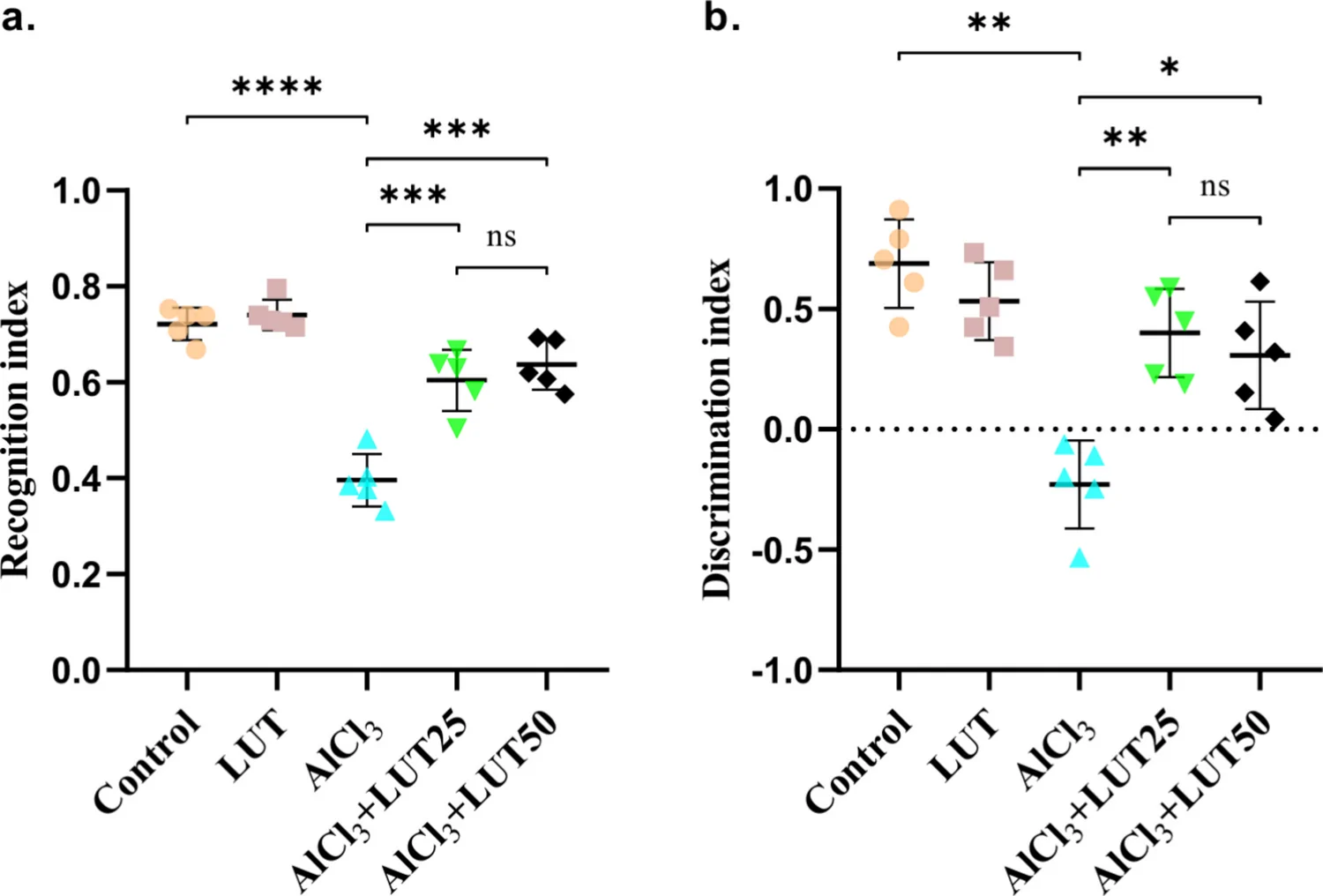
Effects of AlCl_3_ and LUT treatments on recognition memory in the NOR test. **a** RI values, **b** DI scores. All data are expressed as mean ± SEM (*n* = 5 per group). Statistical analysis was performed using one-way ANOVA followed by Tukey’s post hoc test. *p < 0.05, **p < 0.01, ***p < 0.001 vs. AlCl_3_ group; ns, not significant

To investigate the effects of AlCl_3_ exposure and LUT treatment on recognition memory in rats, the NOR test was performed. Memory performance was assessed using both the RI and the DI. The RI value of the AlCl_3_ group was significantly reduced compared to the control group (*p* < 0.0001; Fig. 2a). This deficit was significantly reversed in both AlCl_3_ + LUT25 and AlCl_3_ + LUT50 groups compared to the AlCl_3_ group (*p* < 0.001, both), with no significant difference between them (*p* > 0.05).

Similarly, the DI was markedly decreased in the AlCl_3_ group (*p* < 0.01; Fig. 2b), whereas both LUT-treated groups (AlCl_3_ + LUT25 and AlCl_3_ + LUT50) showed significant improvements compared to the AlCl_3_ group (*p* < 0.01 and *p* < 0.05, respectively), with no significant difference between the two LUT groups (*p* > 0.05).

### LUT modulates Al accumulation in the brains of AlCl_3_-exposed rats

**Fig. 3:**
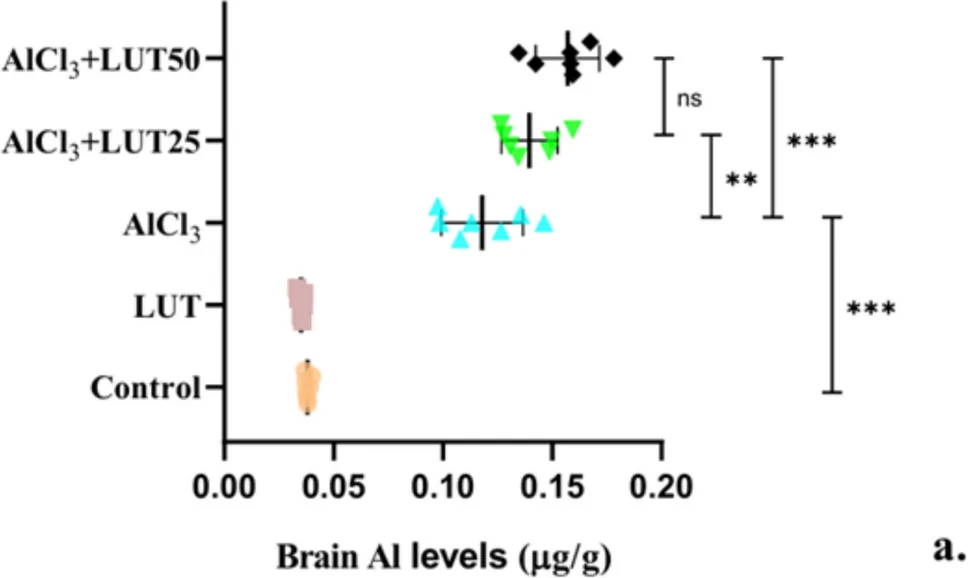
Impact of AlCl_3_ exposure and LUT treatment on brain Al concentrations in rats. Al levels were quantified in whole brain tissue homogenates. Data are presented as mean ± SEM from biologically independent samples (*n* = 7 per group). Statistical analysis was performed using one-way ANOVA followed by Tukey’s post hoc test. **p < 0.01, ***p < 0.001 vs. AlCl_3_ group; ns, not significant

As illustrated in Fig. 3, administration of AlCl_3_ caused a marked elevation of Al concentrations in rat brain tissue compared to the control group, with statistical significance at *p* < 0.001. Interestingly, when LUT was co-administered at doses of 25 mg/kg and 50 mg/kg alongside AlCl_3_, brain Al levels increased even further relative to the AlCl_3_ group, showing significant differences at *p* < 0.01 and *p* < 0.001, respectively. However, no statistically significant difference was observed between the two LUT dosage groups (*p* > 0.05).

### In vitro chelation of Al^3+^ by LUT

**Fig. 4:**
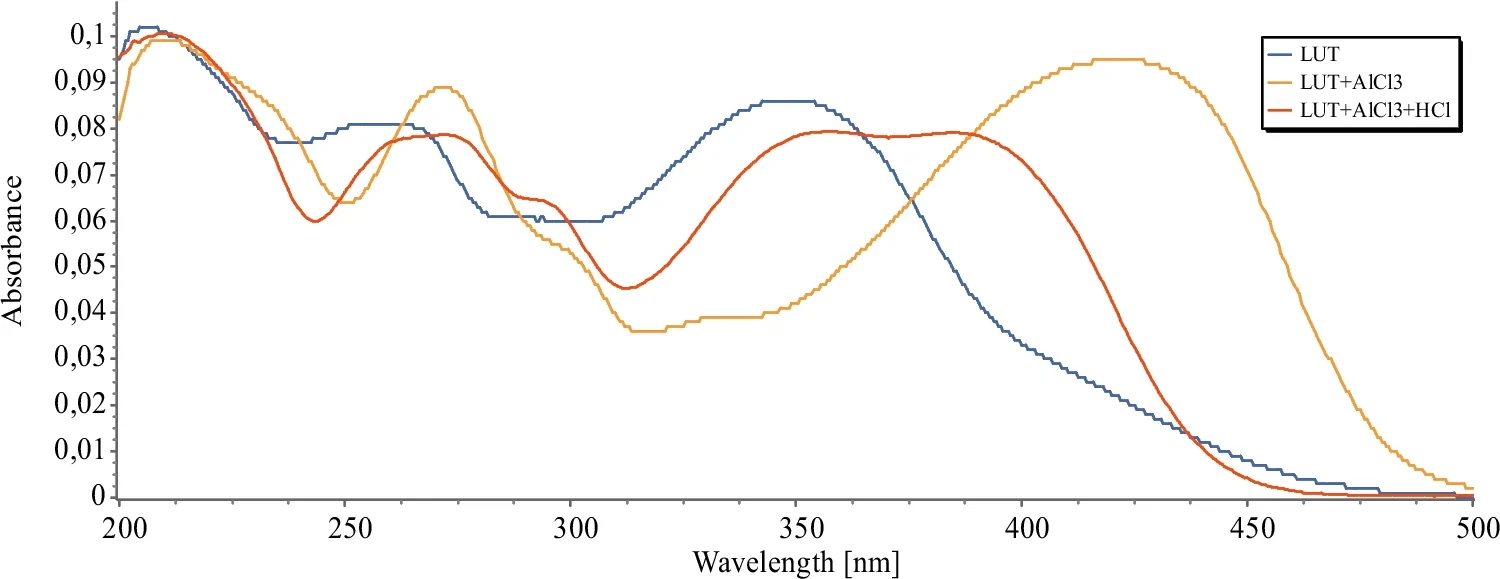
UV–Vis absorption spectra of LUT (blue), LUT in the presence of AlCl_3_ (LUT + AlCl_3,_ orange), and LUT with AlCl_3_ in acidic medium (LUT + AlCl_3_ + HCl, red) recorded in the range of 200–500 nm. The spectral shifts and intensity changes indicate complex formation between LUT and AlCl_3_, as well as the influence of acidic conditions on the stability of the complex

In the current investigation, the UV absorption spectrum of LUT displayed a characteristic maximum absorbance peak at 348.44 nm (represented by the blue curve in Fig. 4). Following the introduction of AlCl_3_ to the solution, a notable redshift was observed, with the absorption maximum shifting to 420.7 nm (depicted by the orange curve). This spectral shift provides strong evidence of a direct interaction and chelation between LUT molecules and Al^3^⁺ ions, resulting in the formation of a LUT–Al^3^⁺ complex. Upon subsequent addition of HCl, the absorption peak exhibited a hypsochromic (blue) shift, moving to 378.46 nm (red curve), suggesting partial dissociation or destabilization of the complex under acidic conditions. These findings collectively indicate that the LUT–Al^3^⁺ chelate maintains structural stability in near-neutral environments but undergoes decomposition or conformational changes when exposed to acidic pH, highlighting the pH-dependent nature of the chelation interaction.

### LUT enhances antioxidant enzyme activities and reduces lipid peroxidation in AlCl_3_-exposed rat brain

Antioxidant enzymes constitute the primary defense mechanism against oxidative stress by neutralizing ROS. To explore the relationship between LUT’s radical-scavenging potential and antioxidant protection, we evaluated lipid peroxidation levels and antioxidant enzyme activities in brain tissue samples.

**Fig. 5:**
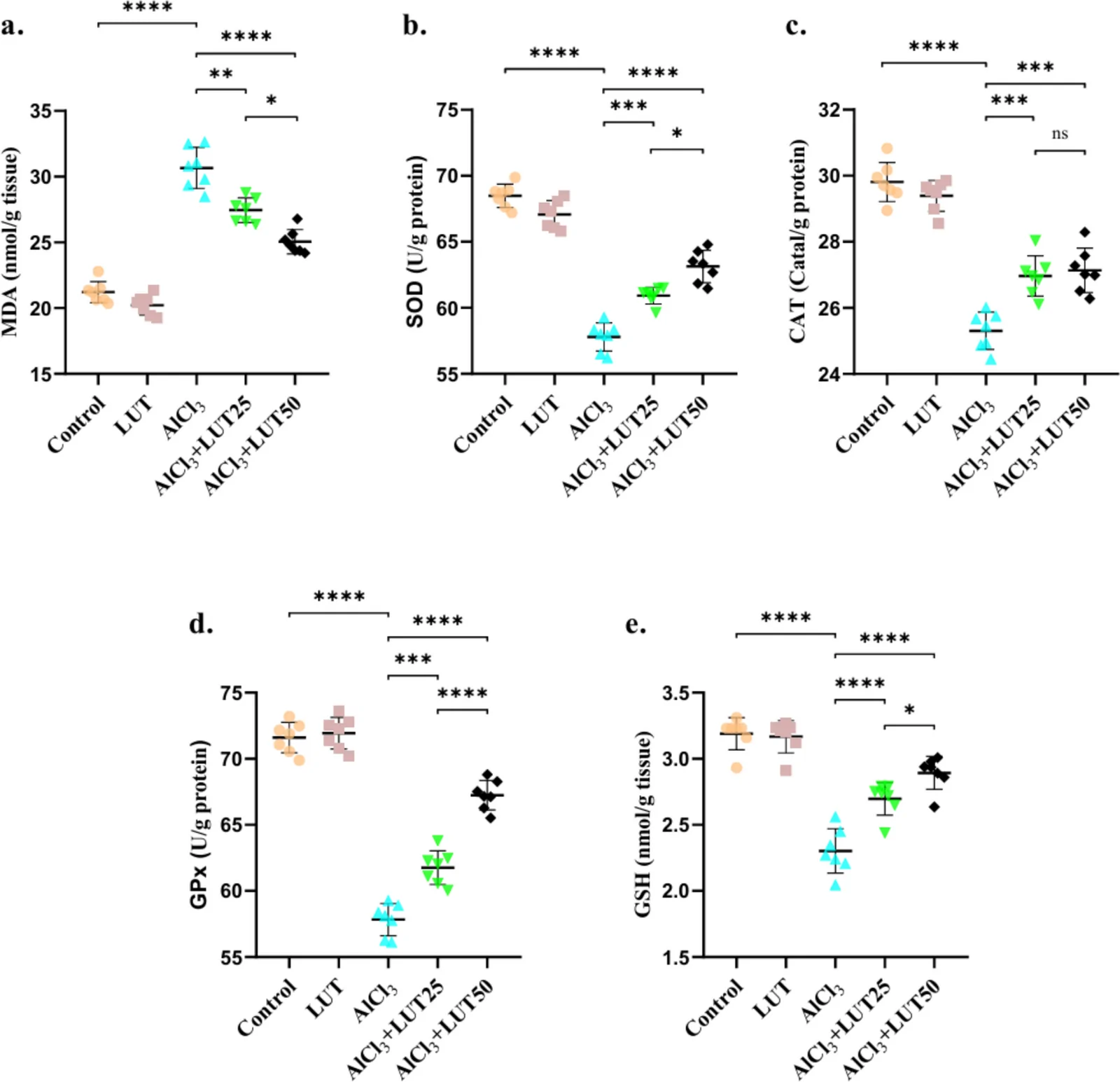
Effects of AlCl_3_ and LUT treatments on oxidative stress biomarkers in rat brain tissue. Bar graphs depict the levels of **a** MDA, **b** SOD, **c** CAT, **d** GPx, and **e** GSH in whole-brain homogenates across experimental groups. Data are presented as mean ± SEM from biologically independent samples (*n* = 7 per group). Statistical comparisons were performed using one-way ANOVA followed by Tukey’s post hoc test. *p < 0.05, **p < 0.01, ***p < 0.001 vs. AlCl_3_ group; ns, not significant

AlCl_3_ exposure resulted in a marked increase in malondialdehyde (MDA) levels compared to the control group (Fig. 5a, p < 0.0001). Treatment with LUT significantly attenuated MDA levels in both the AlCl_3_ + LUT25 and AlCl_3_ + LUT50 groups relative to the AlCl_3_ group (*p* < 0.01 and *p* < 0.0001, respectively). Moreover, the AlCl_3_ + LUT50 group exhibited significantly lower MDA levels than the AlCl_3_ + LUT25 group (*p* < 0.05), suggesting a dose-dependent effect.

AlCl_3_ administration also led to a substantial reduction in the activities of key antioxidant enzymes, superoxide dismutase (SOD, Fig. 5b), catalase (CAT, Fig. 5c), and glutathione peroxidase (GPx, Fig.  5 d), as well as in the levels of the non-enzymatic antioxidant glutathione (GSH, Fig. 5e), all showing high statistical significance (*p* < 0.0001 vs. control). Co-treatment with LUT effectively restored these antioxidant defenses. In the AlCl_3_ + LUT25 group, SOD, CAT, and GPx activities increased significantly (*p* < 0.001), alongside a notable elevation in GSH levels (*p* < 0.0001), compared to the AlCl_3_ group. The AlCl_3_ + LUT50 group showed even greater restoration in antioxidant status, with significantly higher SOD (*p* < 0.0001), CAT (*p* < 0.001), GPx (*p* < 0.0001), and GSH (*p* < 0.0001) levels than the AlCl_3_ group.

Furthermore, when comparing the two LUT-treated groups, the AlCl_3_ + LUT50 group exhibited significantly higher levels of SOD (*p* < 0.05), GPx (*p* < 0.0001), and GSH (*p* < 0.05) than the AlCl_3_ + LUT25 group, although the difference in CAT activity between them was not statistically significant (*p* > 0.05). These findings highlight LUT’s dose-dependent efficacy in mitigating AlCl_3_-induced oxidative damage by enhancing the brain’s antioxidant defense system.

### LUT restores cholinergic function, enhances BDNF expression, and reduces neuronal stress marker c-Fos in AlCl_3_-exposed rat brain

**Fig. 6:**
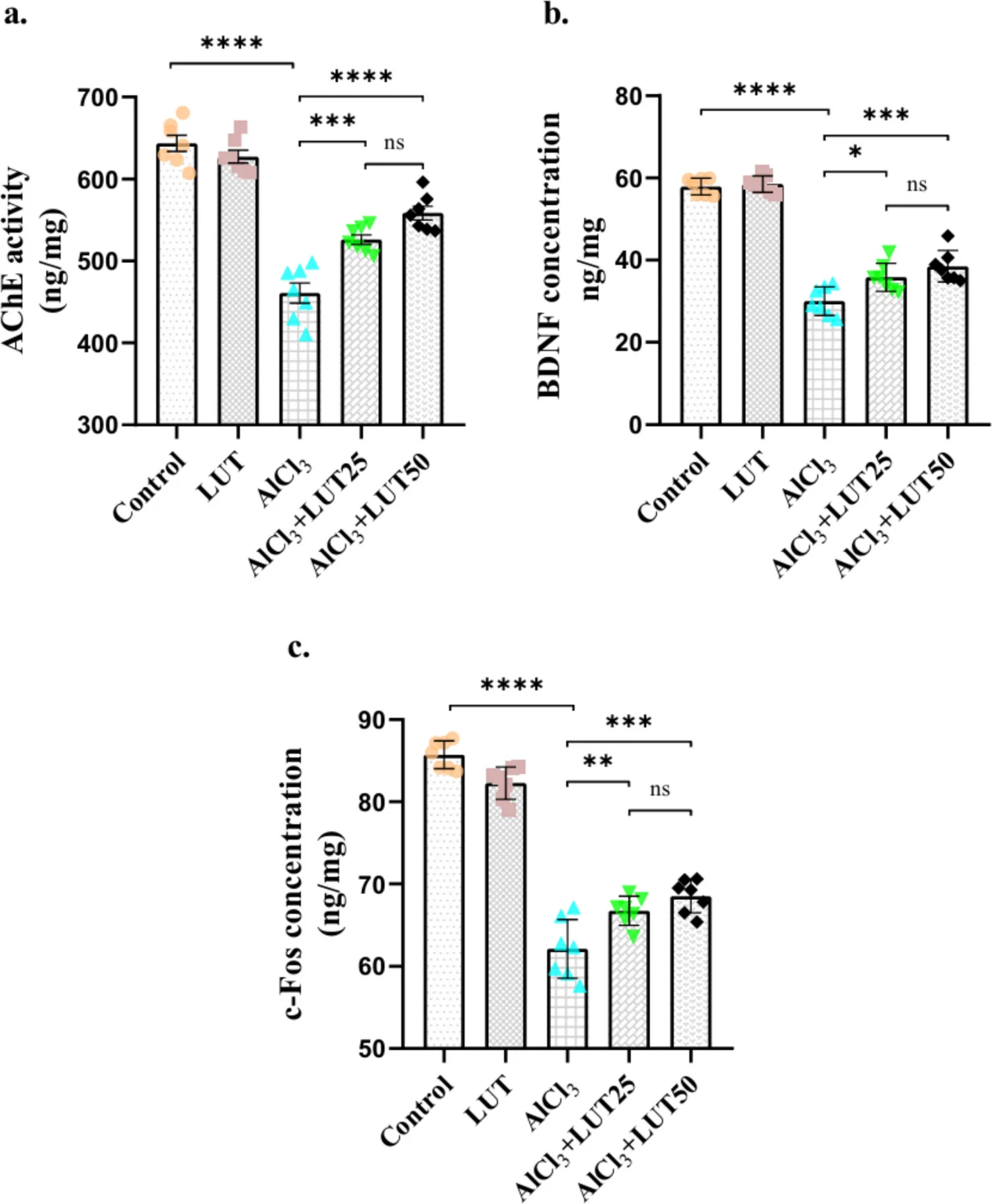
Effects of AlCl_3_ and LUT treatments on neurochemical markers in whole brain tissue. Bar graphs show the levels of **a** AChE activity, **b** BDNF, and **c** c-Fos protein concentration, as determined by ELISA using whole-brain homogenates. Data are presented as mean ± SEM from biologically independent samples (*n* = 7 per group). Statistical analysis was performed using one-way ANOVA followed by Tukey’s post hoc test. *p < 0.05, **p < 0.01, ***p < 0.001 vs. AlCl_3_ group; ns, not significant

AChE is a key enzyme that ends synaptic transmission in the cholinergic system by breaking down acetylcholine. In this study, administration of AlCl_3_ resulted in a pronounced decrease in AChE activity compared to the control group (*p* < 0.0001), indicating a disruption of cholinergic neurotransmission likely linked to neurotoxic effects (Fig. 6a). Treatment with LUT at both 25 mg/kg and 50 mg/kg doses effectively restored AChE activity levels (*p* < 0.001 and *p* < 0.0001, respectively). However, no significant difference was observed between the two LUT treatment groups (*p* > 0.05).

BDNF, an important regulator of neuronal growth, survival, and synaptic plasticity, was markedly decreased after exposure to AlCl_3_ (*p* < 0.0001), which may indicate a mechanism for the neurodegeneration that was seen (Fig. 6b). Both LUT treatment doses enhanced BDNF concentrations significantly, with improvements reaching statistical significance at 25 mg/kg (*p* < 0.05) and more pronounced effects at 50 mg/kg (*p* < 0.001). Nonetheless, the difference between the two doses was not statistically meaningful (*p* > 0.05).

The immediate early gene c-Fos, an established marker of cellular stress and neuronal activation, was evaluated in this study. AlCl_3_ administration resulted in a significant reduction in c-Fos expression (*p* < 0.0001; Fig. 6c), suggesting reduced neuronal activation and impaired neuronal signaling. However, co-treatment with LUT at both 25 mg/kg and 50 mg/kg significantly increased c-Fos levels compared to the AlCl_3_ group (*p* < 0.01 and *p* < 0.001, respectively), suggesting that LUT mitigates Al-induced neuronal stress and excitotoxicity. Notably, no significant difference was observed between the two LUT doses.

### LUT modulates apoptotic markers by decreasing Bax and p53 while enhancing Bcl-2 expression in AlCl_3_-exposed rat brain

**Fig. 7:**
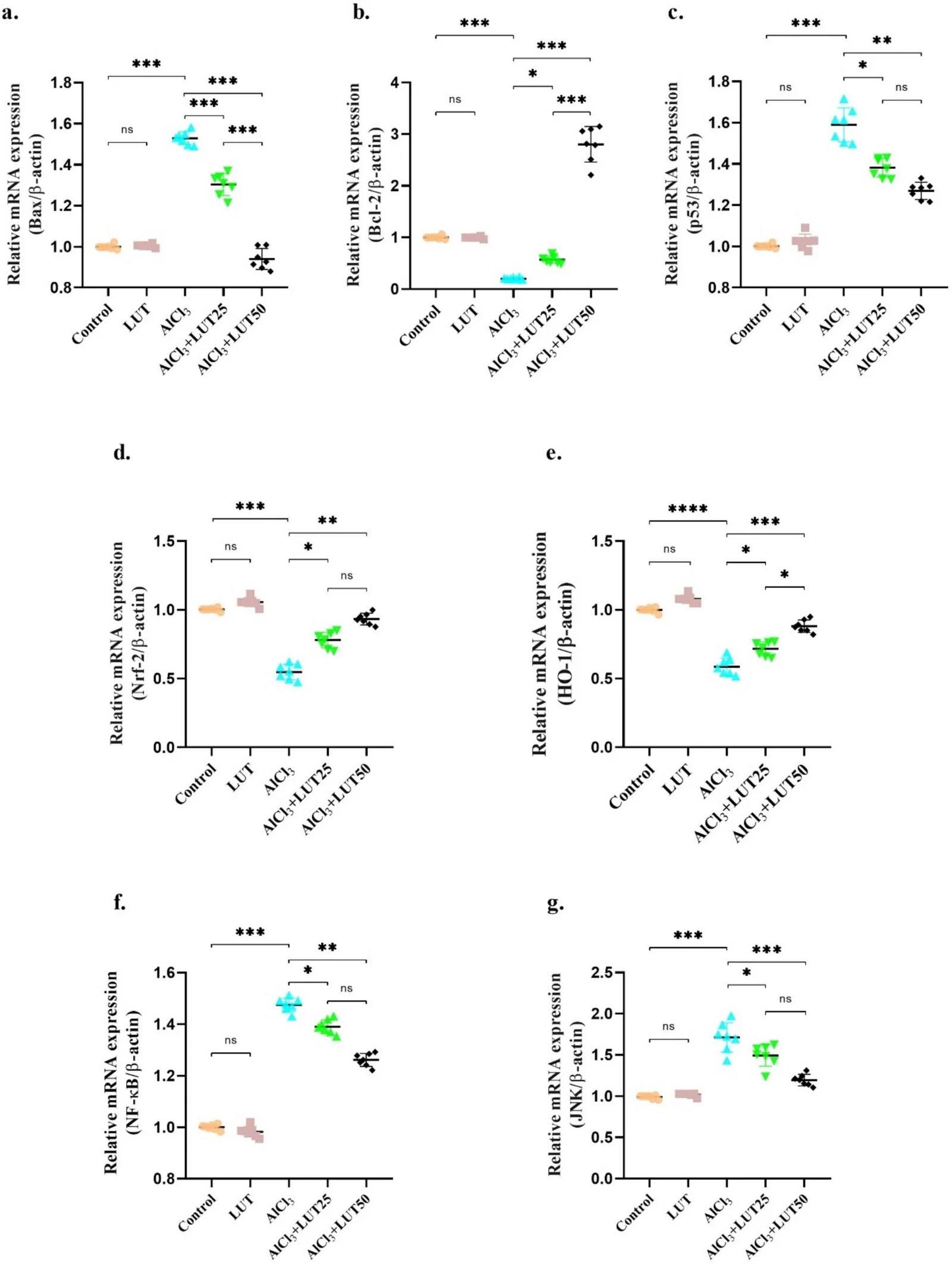
Effects of AlCl_3_ and LUT treatments on the mRNA expression of apoptosis-related genes **a** Bax and **b** Bcl-2 and **c** p53; antioxidant response genes **d** Nrf2, **e** HO-1; and inflammation/stress markers **f** NF-κB and **g** JNK, as determined by qPCR analysis of whole-brain homogenates and normalized to β-actin. Data are presented as mean ± SEM from biologically independent samples (*n* = 7 per group). Statistical analysis was performed using one-way ANOVA followed by Tukey’s post hoc test. *p < 0.05, **p < 0.01, ***p < 0.001, ****p < 0.0001 vs. AlCl_3_ group; ns, not significant

AlCl_3_ administration significantly increased Bax expression compared to the control group (*p* < 0.001, Fig. 7a), indicating enhanced pro-apoptotic signaling. Co-treatment with LUT at both 25 mg/kg and 50 mg/kg significantly reduced Bax expression (*p* < 0.001 for both), suggesting a protective anti-apoptotic effect in a dose-dependent manner (*p* < 0.001).

As shown in Fig. 7b, Bcl-2 expression, a marker of anti-apoptotic activity, significantly decreased in the AlCl_3_ group (*p* < 0.001). LUT treatment reversed this reduction in a dose-dependent manner (*p* < 0.001), with significant increases in the AlCl_3_ + LUT25 (*p* < 0.05) and AlCl_3_ + LUT50 (*p* < 0.001) groups, indicating restoration of anti-apoptotic defenses.

In addition, p53 expression increased significantly in the AlCl_3_ group (*p* < 0.001, Fig. 7c), reflecting cellular stress and potential initiation of apoptosis. LUT co-treatment decreased p53 expression in both the AlCl_3_ + LUT25 (*p* < 0.05) and AlCl_3_ + LUT50 (*p* < 0.01) groups, further supporting its role in mitigating AlCl_3_-induced apoptotic signaling. However, no significant difference was observed between the two doses (*p* > 0.05).

### LUT enhances Nrf2 and HO-1 expression to restore antioxidant defense in AlCl_3_-treated rats

According to RT-qPCR results, rats treated with AlCl_3_ exhibited a marked downregulation of Nrf2 mRNA expression compared to the control group (*p* < 0.001, Fig.  7 d). Importantly, co-treatment with LUT effectively restored Nrf2 expression. Specifically, LUT administration at doses of 25 mg/kg and 50 mg/kg significantly upregulated Nrf2 transcript levels compared to the AlCl_3_ group (*p* < 0.05 and *p* < 0.01, respectively). However, the two doses did not produce significantly different effects (*p* > 0.05).

Consistent with this, HO-1, a key antioxidant enzyme transcriptionally regulated by Nrf2, was also significantly reduced in AlCl_3_-treated rats (*p* < 0.0001, Fig. 7e). LUT co-administration led to a significant elevation in HO-1 expression at both tested doses, with statistical significance at *p* < 0.05 for the AlCl_3_ + LUT25 group and *p* < 0.001 for the AlCl_3_ + LUT50 group. Furthermore, greater efficacy was observed with the 50 mg/kg dose compared to the 25 mg/kg dose (*p* < 0.05).

### LUT reduces AlCl_3_-induced upregulation of NF-κB and JNK

NF-κB, a central regulator of inflammation and immune responses, was markedly elevated in the AlCl_3_ group compared to the control (*p* < 0.001, Fig. 7f). Remarkably, co-treatment with LUT at 25 mg/kg and 50 mg/kg reduced NF-κB transcript levels compared to the AlCl_3_-only group, with statistical significance at *p* < 0.05 and *p* < 0.01, respectively. However, the difference between the two doses was not statistically significant (*p* > 0.05).

In parallel, expression of JNK was significantly upregulated following AlCl_3_ administration (*p* < 0.001, Fig.  7 g). LUT co-administration at both tested doses significantly downregulated JNK expression compared to the AlCl_3_ group (*p* < 0.05 for AlCl_3_ + LUT25 and *p* < 0.001 for AlCl_3_ + LUT50), implying that LUT confers neuroprotective effects by mitigating stress-activated signaling pathways. However, both doses showed similar effects without a significant difference.

### LUT suppresses AlCl_3_-induced upregulation of IL-1β in rat brain tissue

**Fig. 8:**
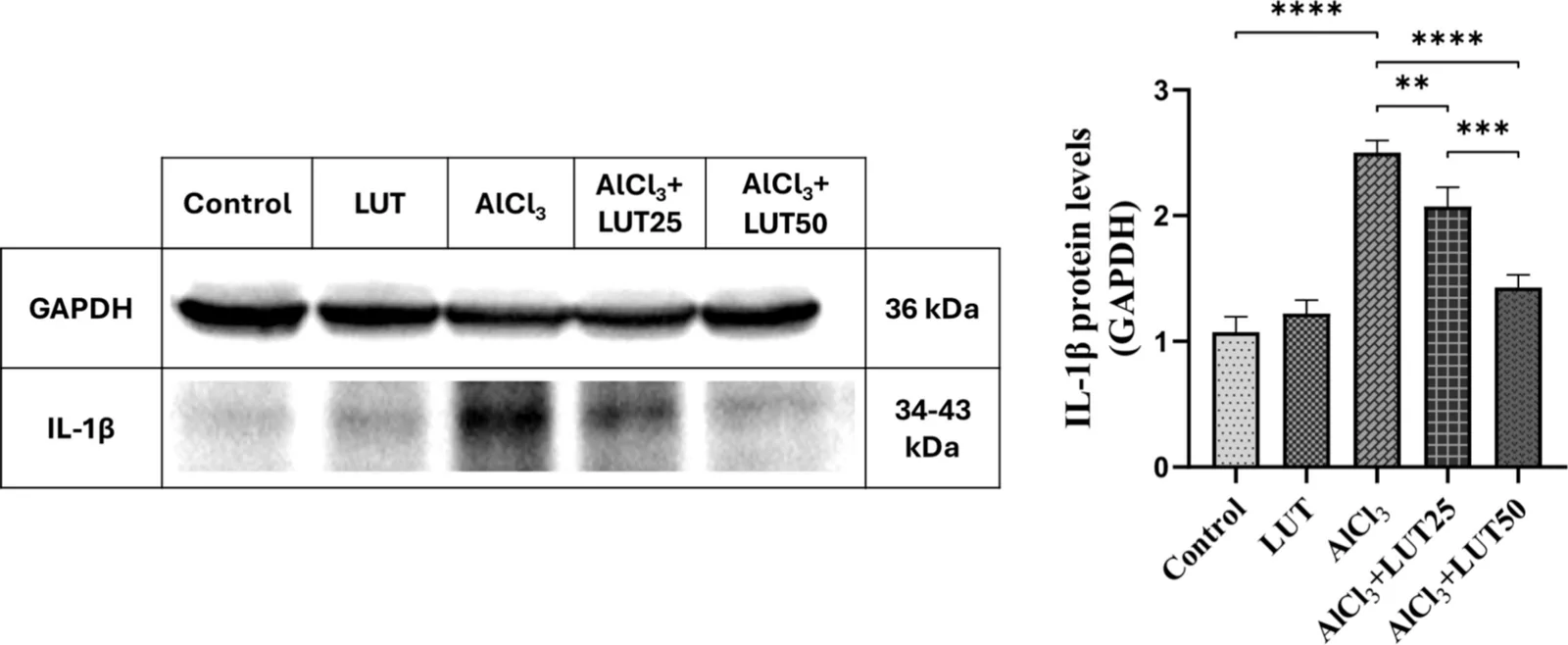
Western blot analysis of IL-1β protein expression normalized to GAPDH in rat brain. *p < 0.05, **p < 0.01, ***p < 0.001, ****p < 0.0001 (one-way ANOVA with Tukey’s test)

Western blot analysis showed that exposure to AlCl_3_ led to a substantial upregulation of IL-1β protein expression in brain tissue compared to the control group (*p* < 0.0001, Fig. 8). Co-administration of LUT at 25 mg/kg significantly reduced IL-1β levels relative to the AlCl_3_-only group (*p* < 0.01), while the higher dose of 50 mg/kg exerted an even more pronounced suppressive effect (*p* < 0.0001).

### Effect of LUT treatment on the immunohistochemistry of rat brain tissues exposed to AlCl_3_

**Fig. 9:**
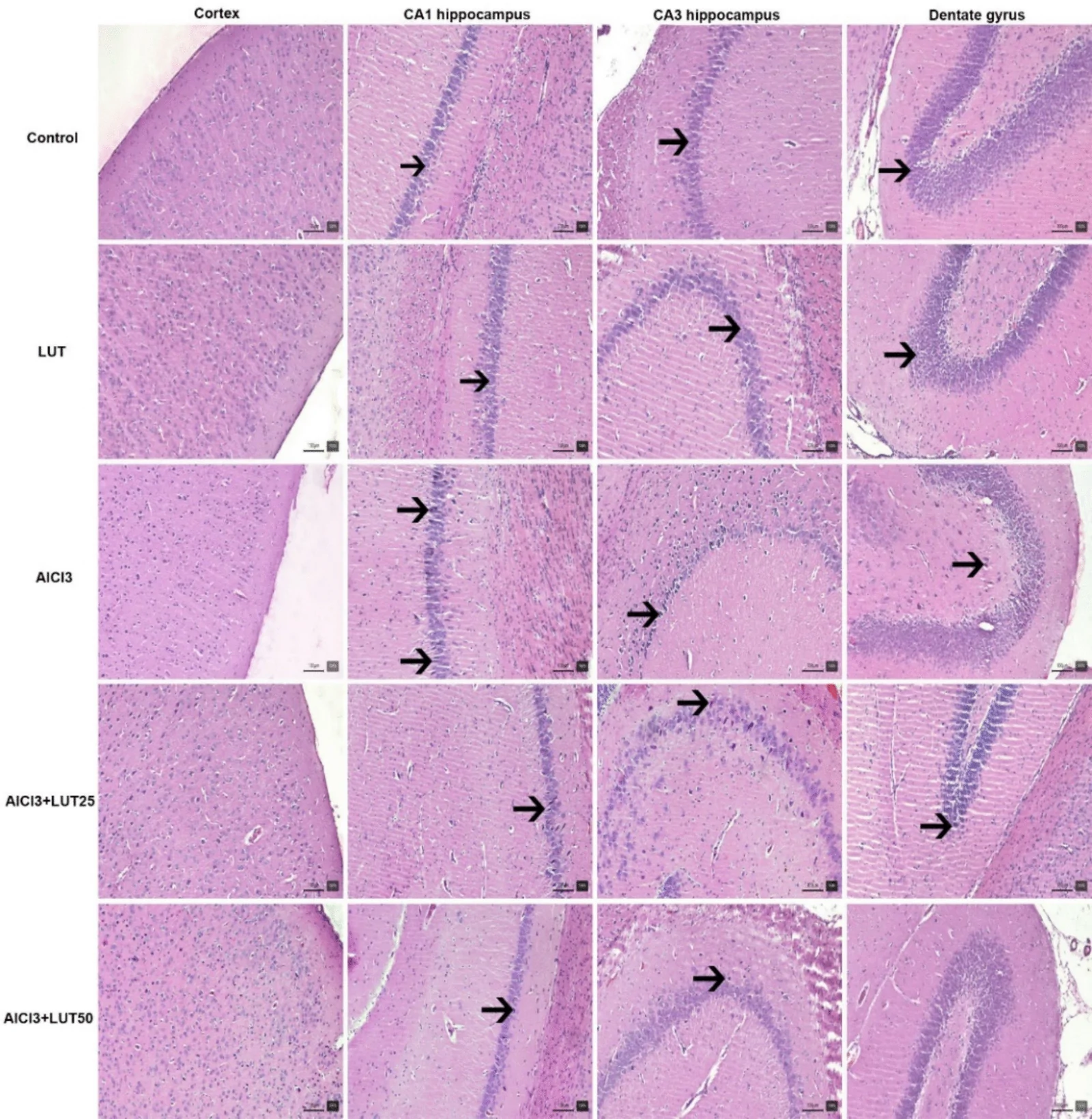
Photomicrographs showing histopathological changes in the cerebral cortex, hippocampal CA1, CA3, and dentate gyrus regions of rats treated with AlCl_3_. H&E staining, × 100 magnification. In the control and LUT-only groups, neurons in the cortex exhibit normal histology, and neurons in the CA1, CA3, and DG regions are lightly stained as indicated by arrows. In sections from the brains of AlCl_3_-treated rats, neuronal degeneration and pyknosis are observed in cortical and hippocampal cells (CA1, CA3, and DG areas), as indicated by arrows. In the AlCl_3_ + LUT25 and AlCl_3_ + LUT50 groups, fewer degenerative neurons with shrunken, darkly stained, pyknotic nuclei are seen in the cortex and hippocampal regions

Figure 9 shows the effect of LUT on histological changes in the cerebral cortex and hippocampal regions in different experimental groups in the AlCl_3_-induced neurotoxicity model. In both the normal control group and the group receiving only LUT, microscopic examination of the cortex and the CA1, CA3, and dentate gyrus regions of the hippocampus revealed no lesions of pathological significance. In the group treated with AlCl_3_, the nuclear membranes in the cortex were indistinct, and the cytoplasm of neurons appeared shrunken and darkly stained. In the same group, neurodegeneration was evident in the hippocampal CA1, CA3, and dentate gyrus regions, characterized by elongated, irregular, dark-stained nuclei and shrunken cytoplasm. In the AlCl_3_ + LUT25 group, the number of dark-stained, shrunken, necrotic neurons in the cortex and hippocampal CA1, CA3, and dentate gyrus regions decreased (Table 2, *p* < 0.05). In the AlCl_3_ + LUT50 group, the number of necrotic neurons in the same brain regions decreased even further compared to the AlCl_3_ + LUT25 group and approached control levels (Table 2, *p* < 0.05).

**Table 2:** Histopathological score of the cerebral cortex and hippocampal region groups/nuclear pyknosis and degeneration

Groups/nuclear pyknosis and degeneration	Cortex	Hippocampal region
Control	0.14 ± 0.14	0.00 ± 0.00
LUT	0.00 ± 0.00	0.00 ± 0.00
AlCl_3_	2.71 ± 0.18*	2.57 ± 0.20*
AlCl_3_ + LUT25	1.57 ± 0.36^#^	1.57 ± 0.29^#^
AlCl_3_ + LUT50	0.42 ± 0.20^&^	0.42 ± 0.20^&^

*p < 0.05 compared to the control group; ^#^*p* < 0.05 compared to the AlCl_3_ group; ^&^*p* < 0.05 compared to the AlCl_3_ + LUT25 group (*n* = 7)

## Discussion

**Fig. 10:**
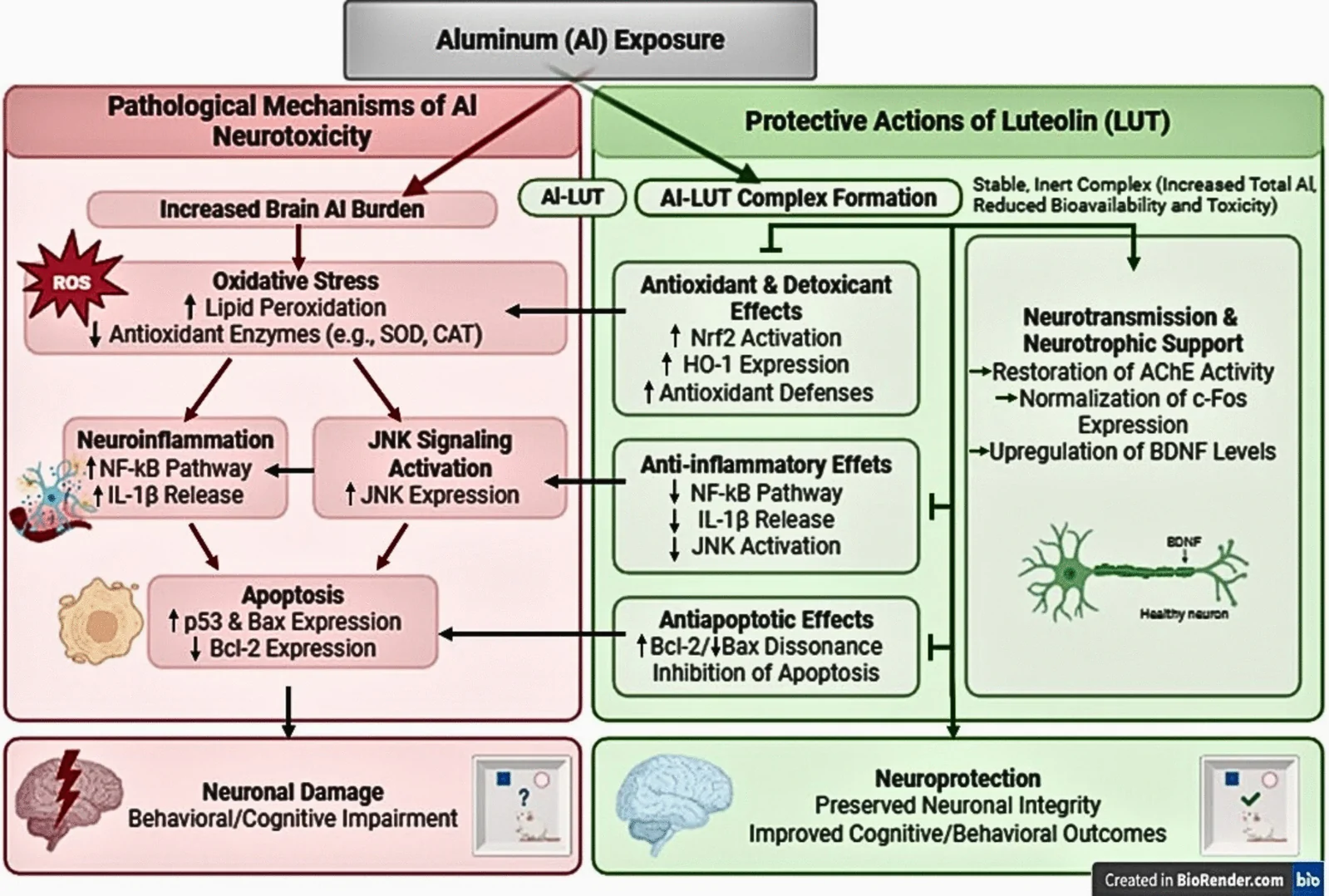
Proposed mechanism of LUT against AlCl_3_-induced neurotoxicity

Aluminum (Al) is a neurotoxic metal associated with the pathogenesis of various neurodegenerative diseases, exerting its harmful effects through the induction of oxidative stress, promotion of neuroinflammation, impairment of neurotransmission, and activation of apoptotic pathways. Given these mechanisms and the widespread exposure to Al, there is increasing scientific interest in identifying effective neuroprotective agents to mitigate Al-induced damage. This study centers on luteolin (LUT), a bioflavonoid widely studied for its ability to protect neural tissue from toxic insults (Jayawickreme et al. 2024). Given its well-documented antioxidant and anti-inflammatory activities (Kempuraj et al. 2021), LUT was evaluated for its potential to mitigate Al-induced neurotoxicity by targeting oxidative stress, inflammation, and apoptosis in the brain. A schematic representation of the mechanistic pathways modulated by LUT in Al-induced neurotoxicity is presented in Fig. 10.

Since Al accumulation is a key contributor to neurotoxicity (Renke et al. 2023), ICP-MS analysis was performed to examine the impact of LUT co-treatment on brain Al levels. As expected, AlCl_3_ exposure markedly increased Al content in brain tissue. Unexpectedly, co-treatment with LUT did not reduce total Al levels; instead, Al concentrations were paradoxically higher in LUT-treated groups. This apparent discrepancy may reflect the formation of stable Al–LUT complexes, which are less bioavailable and less toxic than free Al^3^⁺ ions. Indeed, Malacaria et al. (2023) demonstrated that LUT can chelate toxic metals such as Al(III) and form stable complexes under physiological conditions, and UV-spectrometry findings in the present study further support this interpretation. Because ICP-MS cannot distinguish between bound and free forms (Shang et al. 2013), the measured increase most likely represents complexed species. Therefore, interpreting these data in conjunction with oxidative stress, inflammation, and apoptosis outcomes is essential. Despite higher total Al levels, LUT-treated brains exhibited reduced oxidative stress, neuroinflammation, and apoptotic response, supporting the notion that chelation sequesters Al in a biologically inert form. A similar observation was reported by Li et al. (2017), who showed that Oleracein E (OE) alleviated Al-induced neurotoxicity by reducing oxidative stress and inhibiting apoptosis, despite an increased brain Al concentration in the high-dose OE group. Collectively, these findings highlight the importance of considering both total metal burden and functional outcomes when evaluating neuroprotective interventions.

Here, oxidative stress markers emerge as a central mechanism for interpreting these results, since Al-induced oxidative damage is a major driver of neuronal injury (Laabbar et al. 2021). In the present study, AlCl_3_ administration disrupted redox balance by elevating lipid peroxidation products and suppressing antioxidant enzyme activity, whereas LUT co-treatment effectively maintained redox homeostasis. Although the GSH/GSSG ratio would provide a more precise indicator of redox balance, the combined evaluation of enzymatic antioxidants and lipid peroxidation markers in the present study offers a comprehensive overview of oxidative status. This protective effect is likely mediated through activation of Nrf2, a key transcription factor controlling antioxidant and detoxification responses (Osama et al. 2020). Consistent with previous reports, AlCl_3_ exposure suppressed Nrf2 and HO-1 expression, reflecting weakened endogenous defense mechanisms (Abu-Elfotuh et al. 2025). Notably, LUT treatment reactivated the Nrf2/HO-1 axis, thereby enhancing antioxidant defenses and promoting cellular detoxification. Similar modulation of this pathway has been implicated in neuroprotection across multiple neurodegenerative contexts (Dinkova-Kostova et al. 2018), supporting the interpretation that LUT exerts its beneficial effects, at least in part, through reinforcement of Nrf2-driven antioxidant signaling.

Oxidative stress and neuroinflammation are tightly interconnected processes, and metal exposure, including Al, amplifies this interplay by enhancing ROS production, thereby activating microglia and driving inflammatory responses in the brain (Bondy 2021). NF-κB, a central transcription factor, links oxidative stress to cytokine release by promoting the expression of mediators such as IL-1β, which in turn aggravates neuronal damage through excitotoxicity and synaptic dysfunction (Anilkumar and Wright-Jin 2024). In line with this, our study demonstrated that AlCl_3_ exposure significantly upregulated NF-κB and IL-1β. Importantly, LUT co-administration effectively downregulated both NF-κB and IL-1β, highlighting its anti-inflammatory potential. This effect is likely mediated through antioxidant mechanisms that reduce ROS-dependent NF-κB activation as well as direct inhibition of the NF-κB pathway (Imran et al. 2019). By suppressing glial activation and chronic cytokine release, LUT may help preserve neuroimmune balance and protect against Al-induced neuronal degeneration.

Beyond NF-κB, the JNK pathway also emerged as a critical mediator of Al toxicity. JNK signaling regulates multiple CNS functions, but its persistent activation contributes to neurodegenerative pathology (de los Reyes Corrales et al. 2021; Zhao et al. 2022a). Consistent with previous reports showing enhanced JNK protein expression and phosphorylation in response to Al exposure (Zhang et al. 2020), we observed a significant transcriptional upregulation of JNK mRNA in the brains of AlCl_3_-treated rats. These findings suggest that Al toxicity activates the JNK pathway at both the transcriptional and post-translational levels, thereby amplifying stress-responsive and pro-apoptotic signaling. LUT treatment markedly attenuated JNK expression, in agreement with earlier findings showing that LUT suppresses JNK activation in astrocytes (Che et al. 2020). Taken together, these results suggest that LUT may exert neuroprotection not only by dampening NF-κB/IL-1β-driven inflammation but also by interrupting downstream JNK signaling cascades, thereby limiting stress-induced apoptotic processes.

Apoptosis is a physiological mechanism essential for removing damaged or unnecessary cells and maintaining tissue homeostasis; however, its excessive activation contributes to pathological neuronal loss (Çelik et al. 2020). Consistent with previous studies showing that Al exposure activates apoptotic pathways in the brain (Singla and Dhawan 2015; El-Shetry et al. 2021), our findings demonstrated that AlCl_3_ markedly shifted the apoptotic balance toward cell death, as evidenced by increased expression of p53 and Bax alongside reduced Bcl-2 expression in rat brain tissue. Notably, LUT co-treatment restored the balance of these apoptotic markers, indicating attenuation of Al-induced pro-apoptotic signaling. This modulation of apoptotic pathways suggests that LUT may serve as a promising neuroprotective agent in conditions characterized by dysregulated apoptosis.

Oxidative stress not only drives neuronal injury at the biochemical level but also disrupts neurotransmission (Butterfield and Halliwell 2019), impairs synaptic plasticity (Massaad and Klann 2011), and reduces neurotrophic support such as BDNF signaling (Shen et al. 2023). In this context, our evaluation of brain-derived markers provided complementary insights into how Al toxicity translates into functional neuronal impairment. Consistent with previous reports (Abd El-Aziz et al. 2022), AlCl_3_ exposure significantly reduced AChE activity in our study, indicating impaired cholinergic neurotransmission, which is essential for acetylcholine regulation and for cognitive processes such as learning and memory (Walczak-Nowicka and Herbet 2021).

As an immediate early gene, c-Fos is rapidly upregulated in neurons following physiological stimulation or stress, serving as a marker of neuronal activation and plasticity (Habotta et al. 2022; Chen et al. 2023). A reduction in c-Fos expression may therefore reflect diminished neural excitability, dysfunctional signaling, or even damage to neuronal circuits (Bağcı et al. 2025). Previous studies demonstrated that Al exposure decreased c-Fos gene and protein expression in rat brains, which was linked to learning, memory, and emotional impairments (Liu et al. 2022; Lotfizadeh et al. 2024). In agreement, AlCl_3_ exposure in our study markedly suppressed c-Fos levels, suggesting impaired neuronal activation likely driven by neuroinflammatory and excitotoxic processes.

Finally, BDNF levels were also decreased, consistent with earlier studies showing that Al toxicity reduces neurotrophic support (Liu et al. 2022). As a critical neurotrophin, BDNF supports neuronal survival, differentiation, and synaptic plasticity (Wang et al. 2021), particularly in memory- and learning-related regions such as the hippocampus and cortex (Dadkhah et al. 2023).

Importantly, LUT co-treatment effectively restored AChE activity, normalized c-Fos expression, and upregulated BDNF levels, demonstrating that its neuroprotective actions extend beyond antioxidation. These molecular improvements paralleled the behavioral outcomes, as LUT-treated animals exhibited enhanced cognitive and memory performance compared to the AlCl_3_ group. Consistent with prior studies, Al exposure was associated with decreased locomotor activity, heightened anxiety-like behaviors (Liu et al. 2022; Zghari et al. 2023), and impaired recognition memory (Mehrbeheshti et al. 2022; Dey and Singh 2022b). Taken together, these findings suggest that LUT not only mitigates ROS-mediated cellular damage but also stabilizes cholinergic signaling, preserves neuronal excitability, and enhances neurotrophic support, thereby contributing to improved cognitive and behavioral outcomes under Al-induced neurotoxic conditions.

Histological findings further supported LUT’s neuroprotective effects. While no pathological alterations were observed in the control and LUT-only groups, AlCl_3_ exposure led to marked neuronal damage in both the cerebral cortex and hippocampal regions, including shrunken, dark-stained neurons and disrupted nuclear morphology. Co-treatment with LUT significantly mitigated these changes in a dose-dependent manner. The LUT25 group showed fewer necrotic neurons, and the LUT50 group displayed near-normal histoarchitecture, suggesting that LUT effectively preserves neuronal integrity against AlCl_3_-induced neurotoxicity.

Overall, this study demonstrates that LUT exerts multifaceted neuroprotective effects against Al-induced neurotoxicity. LUT not only attenuates oxidative stress, restores Nrf2/HO-1 antioxidant signaling, suppresses NF-κB/IL-1β-driven neuroinflammation, normalizes JNK activation, and modulates apoptotic pathways, but also preserves cholinergic function and neurotrophic support. Importantly, the neuroprotective effects of LUT occurred despite slightly elevated total brain Al levels, likely due to the formation of stable Al–LUT complexes that reduce the bioavailability and toxicity of free Al^3^⁺ ions, highlighting its chelating potential. These cellular and molecular improvements were reflected in behavioral outcomes and histological analyses, with LUT-treated animals showing improved cognitive performance relative to the AlCl_3_ group and preserved neuronal integrity.

A key finding of this study is the dissociation between total brain Al levels and neurotoxic outcomes. Although LUT-treated groups exhibited higher total Al content, oxidative stress, inflammation, apoptosis, and behavioral deficits were markedly reduced. This paradox suggests that total metal burden may not reflect toxic bioavailability and supports the formation of less reactive LUT–Al complexes. Together, these findings indicate that LUT-mediated neuroprotection may involve modulation of Al bioavailability, in addition to its classical antioxidant and anti-inflammatory actions. This integrative approach, combining metal quantification, chelation evidence, and multi-pathway molecular analysis, provides mechanistic insight into potential flavonoid-mediated protection against metal-induced neurotoxicity.

While the present findings provide comprehensive evidence of LUT-mediated neuroprotection against Al-induced brain injury, several limitations should be acknowledged. The study employed a whole-brain approach, which, although suitable for assessing global neurotoxicity, does not allow region-specific or cell-type–resolved mechanistic analysis. Future investigations incorporating hippocampal and cortical dissections, as well as neuron- and glia-specific assessments, would provide deeper insight into localized signaling alterations. Moreover, additional studies are warranted to validate these findings in larger and more representative experimental models, to clarify the pharmacokinetic profile of LUT under chronic exposure conditions, to optimize dosing strategies, and to evaluate long-term safety. Assessing the translational applicability of LUT in clinical settings, particularly in populations with chronic environmental Al, represents an important direction for future research.

## Conclusion

This study demonstrates the neuroprotective potential of LUT against AlCl_3_-induced neurotoxicity. LUT mitigated oxidative stress through activation of the Nrf2/HO-1 axis, suppressed NF-κB/IL-1β-mediated neuroinflammation, restored apoptotic balance, and improved neuronal and behavioral outcomes. Notably, the protective effects occurred despite elevated total brain Al levels, suggesting that LUT may modulate Al bioavailability in addition to exerting antioxidant and anti-inflammatory actions. Collectively, these findings position LUT as a promising candidate for mitigating neurodegeneration associated with chronic environmental metal exposure. Nevertheless, further studies addressing region-specific mechanisms, pharmacokinetics, optimal dosing, and long-term safety are necessary to strengthen its translational relevance.

## Supplementary Information

Below is the link to the electronic supplementary material.

ESM1 (PDF 237 KB)

ESM 1 (PNG 110 KB)

High resolution image (TIF 2.68 MB)

ESM 1 (PNG 110 KB)

High resolution image (TIF 2.68 MB)

## Data Availability

The datasets generated during and/or analyzed during the current study are available from the corresponding author upon reasonable request.
