# Supplementary material for: Neuroprotective effects of luteolin against aluminum-induced brain injury via chelation and antioxidant pathways
Source: Naunyn Schmiedebergs Arch Pharmacol. 2026 May 27;399(11):17715–33. doi: 10.1007/s00210-026-05459-7 (PMC13503386; doi:10.1007/s00210-026-05459-7)
Supplement: Supplementary file 1 [file 210_2026_5459_MOESM1_ESM.pdf]

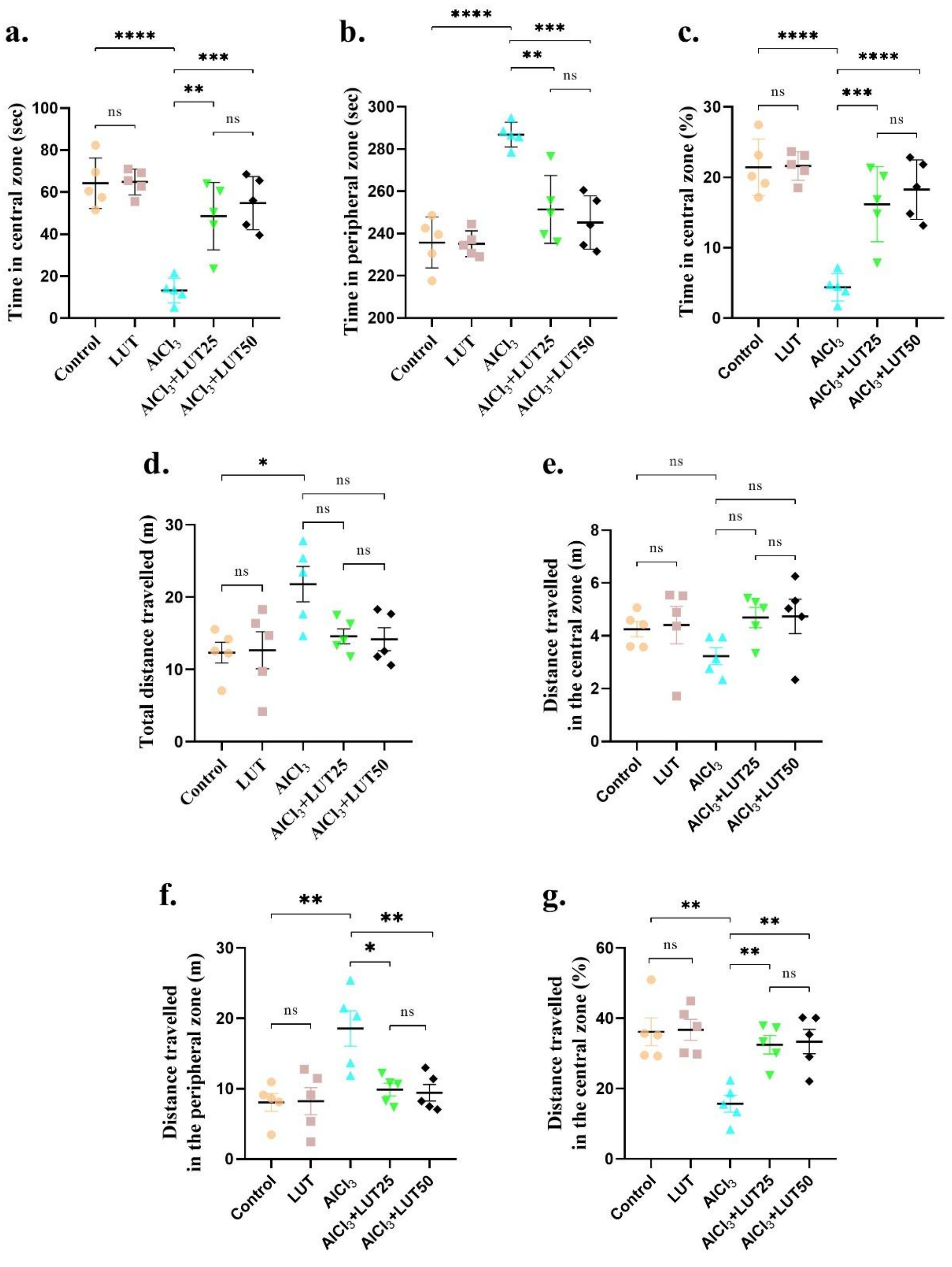

Supplementary Figure 1. Effects of $AlCl_3$ and LUT treatments on open field test parameters. Open field behavioral performance of rats across experimental groups. Graphs show (a) time spent in the central zone (s), (b) time spent in the peripheral zone (s), (c) percentage of time spent in the central zone (%), (d) total distance travelled (m), (e) distance travelled in the central zone (m), (f) distance travelled in the peripheral zone (m), and (g) percentage of distance travelled in the central zone (%). Data are presented as mean ± SEM from biologically independent animals (n = 5 per group). Statistical analysis was performed using one-way ANOVA followed by Tukey's post hoc test. $^{**}p < 0.05$, $^{**}p < 0.01$, $^{***}p < 0.001$, $^{****}p < 0.0001$ vs. $AlCl_3$ group; ns: not significant.
